# Psychometric Properties of the Interpersonal Needs Questionnaire-15 in Spanish Adolescents

**DOI:** 10.3389/fpsyt.2022.833400

**Published:** 2022-03-08

**Authors:** Sandra Pérez Rodríguez, Joaquín García-Alandete, Blanca Gallego Hernández de Tejada, Verónica Guillén, Jose Heliodoro Marco

**Affiliations:** ^1^Department of Personality, Evaluation and Psychological Treatment, Faculty of Psychology, University of Valencia, Valencia, Spain; ^2^Department of Personality, Assessment and Therapeutic Intervention, Catholic University of Valencia, Valencia, Spain; ^3^Ciber Fisiopatología Obesidad y Nutrición (CIBEROBN), Madrid, Spain

**Keywords:** adolescents, psychometric properties, Interpersonal Needs Questionnaire-15 (INQ-15), Spanish, multi-group confirmatory factor analysis

## Abstract

**Background:**

Thwarted Belongingness (TB) and Perceived Burdensomeness (PB) are considered risk factors of suicide behavior in the Interpersonal Theory of Suicide and constitute the main factors of the Interpersonal Needs Questionnaire—INQ.

**Aims:**

The present study analyzes the internal consistency, construct validity, and invariance across sex and age of the INQ-15, which comprises two subscales, in a sample of Spanish community adolescents.

**Methods:**

Participants were 1,536 adolescents from 12 to 19 years old. The INQ-15, the total number of non-suicidal self-injuries (NSSI), the Hopelessness Scale, and the Purpose in Life Test-Adolescents (PIL-A) were used.

**Results:**

The INQ-15 showed good internal consistency for TB ($\bar{\omega }$ = 0.88) and PB ($\bar{\omega }$ = 0.78) subscales and construct and concurrent/discriminant validity in the whole sample. Both the PB and TB subscales showed a good fit {$SB{\chi^{2}}_{\left(9\right)}$ = 6.448, *p* = 0.694, CFI = 1.000, RMSEA = 0.000 [90% CI (0.000, 0.022)] and $SB{\chi^{2}}_{\left(27\right)}$ = 248.973, *p* = 0.000, CFI = 0.922, RMSEA = 0.073 [90% CI (0.065, 0.082)]}, respectively. Regarding the invariance analyses, we found (1) non-invariance in the PB subscale across sex groups and metric, scalar, and stric invariance across age groups, and (2) that it was not possible to perform the invariance analysis for the TB subscale across both sex and age because the fit was not adequate for both boys and 12–15 years old groups. Positive and significant relationships were found between the INQ-15 subscales and hopelessness and NSSI frequency, and negative and significant correlations with meaning in life.

**Conclusions:**

The INQ-15 is a valid instrument for assessing TB and PB in Spanish adolescents. Future studies should analyze the invariance of this instrument in adolescents across sex and age.

## Introduction

### Suicide, Perceived Burdensomeness, and Thwarted Belongingness

Suicide is a serious global public health issue (1). In the year 2018 in Spain (2), 3,679 people ended their lives, making suicide the leading non-natural cause of death and the second cause of death in adolescents and young adults. Literature on suicide has shown that about one-third of people with suicide ideation attempt suicide (3, 4), and the same trend has been found in adolescents (5). For this reason, some authors (6, 7) have highlighted that it is crucial to identify the factors that lead to developing suicide ideation and those that drive people to make a suicide attempt.

From the ideation to action framework, the Interpersonal Theory of Suicide (IPTS) (8, 9) posits that both Perceived Burdensomeness (PB: the perception of being a burden to others) and Thwarted Belongingness (TB: loneliness and the perception of not receiving any kind of reciprocal care) are necessary and independent factors in developing passive suicide ideation (desire for death). Some empirical evidence supports the links between PB, TB, and suicide ideation [e.g., (6, 10, 11)]. To develop active suicide ideation, the person needs to think that these two states will not change in the future, thus leading to hopelessness (9). Finally, to reach suicidal behavior, according to the IPTS, the patient must acquire the capacity for suicide through fearlessness toward death and increased pain tolerance, which, in turn, develop through repeated experiences of painful events, such as exposure to violence. A broad subset of research has supported the theory in adults [e.g., (12)] and adolescents [e.g., (13–15)].

### A Scale for Assessing Perceived Burdensomeness and Thwarted Belongingness: The Interpersonal Needs Questionnaire

To test the aforementioned constructs of PB and TB, the Interpersonal Needs Questionnaire (INQ-25) was developed for use by researchers in the investigation of the etiology of suicidal behavior, as well as by clinicians as part of a risk assessment framework (6). This scale includes 25 items rated on a 7-point Likert type scale (0 = Not at all true for me; 7 = Very true for me), with 10 items assessing PB and 15 assessing TB. In order to reduce the administration time and improve multicollinearity (16), different INQ versions have been proposed.

A 12-item version (7 items for PB, 5 for TB) (16) was developed, for which the authors found adequate internal consistency indexes: TB, α = 0.85, PB, α = 0.89. Freedenthal et al. (17) carried out a confirmatory factor analysis of the INQ-12 in a sample of adult American undergraduate students, confirming both factors. Internal consistency was above 0.90 for both subscales, and both had adequate convergent validity.

An alternative 18-item version was published (6), and Marty et al. (18) explored its psychometric properties in a sample of American community older adults. A principal axis factor analysis showed two factors consistent with the PB and TB constructs and both PB and TB provided evidence for convergent validity. However, in this work, the authors did not carry out a confirmatory factor analysis of the instrument.

Van Orden et al. (19) explored the psychometric properties of the INQ-25 in five different samples of American undergraduate students, adult outpatients, and healthy older adults; after analyzing its factorial structure, they proposed a version with 15 items; 6 items assessing PB (items 1–6) and 9 assessing TB (items 7–15). Items 7, 8, 10, 13, 14, and 15 are reverse-worded and scored. Similar results were found using this version in a sample of American military personnel (20). Results from the multiple group CFA confirmed a two-factor structure and its invariance across the different groups. Convergent and divergent validity were found for PB, but divergent validity was not found for TB.

A multicentric study carried out with Hispanic participants used a Spanish translation of the INQ-15 (21) in three samples of American young adults, Mexican patients and young adults, and Spanish college students. The authors found that the 15-item version did not adequately fit the data from the three samples, and that a 9-item 2-factor solution provided the best fit. Both subscale scores demonstrated good internal consistency, 1-week test-retest reliability, and convergent validity. In addition, the instrument also showed measurement invariance across nationalities and clinical severity in the three samples. However, low divergent validity was found. Teo et al. (22) confirmed the two-factor structure of the INQ-15 in young adults in Singapore with good internal consistency, concurrent, convergent, predictive, and discriminant validity.

Moreover, it has been found that cut-off scores of the INQ-15 of 17 for PB and 37 for TB correctly classificated high-risk and low-risk women with chronic illnesses, thus supporting the clinical relevance of the instrument (23). Mitchell et al. (24) also found INQ-15 as a useful instrument to predict suicide ideation-related outcomes in psychiatric inpatients.

A study explored the structure of the INQ-15 in a German adult community and a clinical sample (25). The results revealed that the PB scale showed good fit in the clinical sample, but not in the general population. In addition, a TB 5-item version fit the clinical sample, but not the general population. In addition, Wang et al. (26) found adequate psychometric properties of the INQ-15 in Chinese migrant industrial workers, finding that predicted adequately suicide ideation in this population.

The IPTS has obtained growing evidence for its validity in adolescent samples (27). These authors highlighted the need to validate the IPTS constructs in adolescence, given that some items validated in adults “might need to be changed or supplemented to capture the relevant constructs in the context of adolescence” [(27), p. 8] because adolescence is a developmental stage that involves great emotional instability and can affect psychological adjustment (28). In addition, adolescence has been found to be a period when the risk of engaging in self-injurious behaviors increases [i.e., (29)]. However, few studies have explored the psychometric properties and factorial structure of the INQ (19) in community adolescents. Among them, Hill et al. (30) compared the psychometric properties of the different INQ versions, including the INQ-15 (10, 12, 15, 18, and 25 items) (6, 17, 18, 31), in three sample of American college students and adolescents from an inpatient unit. The authors reported acceptable psychometric properties for all the INQ versions but highlighted the best fit for the 15- and 10-item versions, thus recommending them for future research. In all the samples, good internal consistency was found for both subscales, as well as adequate concurrent predictive validity of suicidal ideation.

Podlogar et al. (32) translated and validated the INQ-15 in a sample of 307 Slovenian 12 to 17-year-old adolescents, confirming a 12-item version and two-factor solution (TB and PB) with six items in each factor. In addition, the authors found adequate concurrent and divergent validity and stability across a period of 7 months.

In a recent study, El Behadli et al. (33) explored the structure of the INQ-25 in American adolescents, reducing the scale to 10 items, five per scale. Large correlations were found between the original scale and the new scale, along with good reliability and validity indexes. And finally, a recent study (34) compared five versions of the INQ in Chinese adolescent samples, finding that the INQ-15, the INQ-12 and the INQ-10 were the most suitable versions for teenagers (12–18 years).

In sum, an increasing number of studies are analyzing the psychometric properties, factorial structure, and clinical utility of the different versions of the INQ. Nevertheless, there is high heterogeneity in the analyzed versions across studies, and some of them have identified limitations mainly related to high intercorrelations between subscale scores or low divergent validity for the TB subscale (19, 21). As Podlogar et al. (32) pointed out, the INQ-15 is the first empirically derived and psychometrically validated version of the original version of the INQ. To our knowledge, to date only two studies have explored the psychometric properties of the INQ-15 in adolescents: Hill et al. (30) in American clinical adolescents and Podlogar et al. (32) in Slovenian community adolescents. In addition, although Silva et al. (21) recently proposed a Spanish translation for different Hispanic samples, no studies have translated the INQ-15 and explored its factorial structure in Spanish adolescents, and there is still a need for instruments to screen risk factors for suicide in Spain.

### The Present Study

The main objective of the present study was to translate the INQ-15 into Spanish and confirm its internal consistency and two-factor structure in a Spanish sample of community adolescents between 12 and 19 years old. Moreover, we aimed to analyze the invariance of the INQ-15 across sex and age (12–15 and 16–19 years) and explore the validity of the scale. We expected to find statistically significant positive correlations between the PB and TB subscales and non-suicidal self-injury (NSSI) frequency and hopelessness, and negative correlations with meaning in life.

## Methods

### Procedure and Participants

The study procedure was approved by the ethical committees of the university. A university professor of Scientific English and a member of the research team translated the ISAS to Spanish. A synthesis of both versions was carried out solving the discrepancies, and then two experts English-Spanish translators carried out a back-translation. Three members of the research team independently reviewed the final version of the instrument, following directions proposed by Arafat et al. (35). We carried out a pilot study with 50 students to improve the questionnaire's administration and the wording of the instrument.

Researchers on the team contacted the principals of 22 schools and high schools in different areas all over Spain. Nine agreed to participate in the study. Students were recruited through classroom announcements, and consent letters were sent home by the administrators of the schools. Participation was voluntary and anonymous, and participants did not receive any compensation for participating in this study. We followed the World Health Organization (36) definition of adolescents as people from 10 to 19 years old. The inclusion criteria were: males or females between 12 and 19 years old (lower secondary and—high school in Spain) and provide their informed consent and/or that of their parents. The exclusion criteria were the refusal of the students and/or their parents to participate in the study and being under 12.

The study sample, composed of students from different Spanish provinces, was recruited between September 2016 and June 2018. A total of 1,733 adolescents were initially approached, of whom 193 (11.16%) were not included: 22 (1.27%) refused to participate in the study or did not obtain their parents' consent, and 171 (9.89%) did not completely fulfill the assessment protocol.

Therefore, a total of 1,536 participants between 12 and 19 years old, *M* = 14.87, *SD* = 1.58, were included in the study and assessed on their socio-demographic characteristics and a subset of psychological variables that were analyzed in a broader NSSI risk-factor study. The boys' group included 739 (48.11%) participants, and the girls' group included 797 (51.89%) participants. A total of 985 (64.13%) adolescents were 12–15 years old, and 551 (35.87%) were 16–19 years old. For the data collection, students filled in online questionnaires, always with the help of one or two members of the research team (Table 1).

**Table 1:** Sociodemographic variables.

***N* = 1,536**	***n* (%)**	**Range**
Age *M* (*SD*)	14.87 (1.58)	12–19
Men	739 (48.11)	
Women	797 (51.89)	
12–15 years old	985 (64.13)	
16–19 years old	551 (35.87)	

### Instruments

#### Interpersonal Needs Questionnaire-15 Items

The INQ-15 (19) is a self-report measure composed of 15 items that evaluate the main constructs of interpersonal suicide theory (8): PB (items 1–6), and TB (items 7–15). The items are answered on a 1–7 Likert scale. In this study, both the PB and TB dimensions showed high and acceptable internal consistency, $\bar{\omega }$ = 88 and $\bar{\omega }$ = 78, respectively.

#### Purpose in Life-Adolescents

The Purpose in Life-Adolescents (37) is a 9-item adaptation of the Spanish version of (38) PIL (39) for assessing meaning in life in adolescents. The PIL-A is answered on a Likert scale (1–7). In this study, the PIL-A showed a good fit, *SB*χ^2^_(26)_ = 47.157, *p* = 0.007, CFI = 0.997, RMSEA = 0.023 [90% CI (0.012, 0.033)] and high internal consistency, $\bar{\omega }$ = 0.89.

#### Inventory of Statements About Self-Injury [ISAS Part I]

The presence of NSSI was evaluated using the ISAS (40) part I of the ISAS. The ISAS-I asks about the lifetime frequency of 12 different NSSI behaviors performed intentionally and without suicidal intent (e.g., banging/hitting self, biting, burning, carving, and cutting). Adolescents were asked how many times they had injured themselves.

#### Beck Hopelessness Scale

The Beck Hopelessness Scale (41) is a 20-item scale designed to assess negative expectations about the future. It has high internal consistency, α = 0.93, and it has also been validated in a Spanish sample (42). In our sample, the BHS showed a good fit, *SB*χ^2^_(167)_ = 196.059, *p* = 0.062, CFI = 0.996, RMSEA = 0.011 [90% CI (0.000, 0.016)] and good internal consistency, $\bar{\omega }$ = 0.84.

### Statistical Analyses

First, the means (and standard deviations), skewness and kurtosis (and standard errors), Kolmogorov-Smirnoff normality test, and corrected item-total correlations of the INQ-15 items and subscales were calculated. A Confirmatory Factor Analysis (CFA) was carried out to evaluate the structural invariance of the INQ-15. Because data distribution was not normal, the Robust Maximum Likelihood estimation (43) was used. Likewise, given that the INQ-15 is an ordinal scale, the Diagonally Weighted Least Squares (DWLS) method was used. Fit indices included the Comparative Fit Index (CFI; a value ≥0.90 indicates acceptable fit and a value ≥0.95 indicate good model fit) and the Root Mean Square Error of Approximation (RMSEA; a value <0.080 indicates acceptable model fit and a value <0.050 indicates good model fit) [e.g., (44)]. The correlation between the PB and TB subscales was assumed when performing the INQ-15 CFA in the whole sample, the boys' and girls' groups, and the 12–15 and 16–19-year-old groups.

Increasingly restrictive models were iteratively examined to determine the degree of model invariance across sex (boys/girls) and age (12–15 years/16–19 years) (45). To evaluate the fit difference between nested models, the differences between the CFI fit index (ΔCFI) and the RMSEA index (ΔRMSEA) were used: Values ≤ 0.01 on ΔCFI (46) and an increase <0.015 on the ΔRMSEA (47) indicate invariance.

The convergent validity of the INQ-15 was reported using the correlation with the number of NSSI and the HS scale, and the discriminant validity was obtained using the correlation with the PIL-A, a measure of meaning in life that has been related to PB and TB (18). These correlations were computed using Spearman's *rho* (ρ), the differences between boys' and girls' correlations were calculated (48), and effect-sizes (49) were reported.

The free software JASP for Windows (50) was used to carry out these statistical analyses.

## Results

### Descriptive Statistics of the Scales Used in the Present Study

Table 2 shows the descriptive statistics of the INQ-15 items and both the PB and TB dimensions of the INQ-15, as well as the corrected item-total correlations. Data distribution was not normal: the skewness value for items 1–5, 7, and 13, as well as for the PB subscale, was > 2, and the kurtosis value for items 4 and 5 was > 7 (51). The Kolmogorov-Smirnov normality test was significant for all the INQ-15 items and subscales. The correlation between the PB and TB subscales, ρ = 0.51, was assumed when performing the CFA of the INQ-15.

**Table 2:** Descriptive statistics of the items and subscales of the INQ-15.

**INQ-15**	***M (SD)***	**Skewness (*SE*)**	**Kurtosis (*SE*)**	**Kolmogorov-Smirnov** **normality test^a^**	**Corrected item** **total correlation**
Item 1	1.668 (1.364)	**2.011 (0.062)**	3.420 (0.125)	0.462^**^	0.841^**^
Item 2	1.613 (1.330)	**2.190 (0.062)**	4.263 (0.125)	0.471^**^	0.858^**^
Item 3	1.577 (1.333)	**2.429 (0.062)**	5.475 (0.125)	0.475^**^	0.768^**^
Item 4	1.269 (0.966)	**4.037 (0.062)**	**17.145 (0.125)**	0.522^**^	0.739^**^
Item 5	1.370 (1.074)	**3.156 (0.062)**	**10.151 (0.125)**	0.507^**^	0.734^**^
Item 6	2.249 (1.737)	1.144 (0.062)	0.367 (0.125)	0.365^**^	0.768^**^
Item 7	1.502 (1.202)	**2.493 (0.062)**	5.924 (0.125)	0.485^**^	0.555^**^
Item 8	2.610 (1.846)	0.775 (0.062)	−0.375 (0.125)	0.306^**^	0.698^**^
Item 9	2.463 (2.206)	1.177 (0.062)	−0.161 (0.125)	0.381^**^	0.397^**^
Item 10	1.850 (1.588)	1.805 (0.062)	2.385 (0.125)	0.439^**^	0.659^**^
Item 11	2.257 (1.763)	1.148 (0.062)	0.323 (0.125)	0.368^**^	0.719^**^
Item 12	2.206 (1.756)	1.232 (0.062)	0.539 (0.125)	0.379^**^	0.635^**^
Item 13	1.674 (1.446)	**2.167 (0.062)**	3.962 (0.125)	0.465^**^	0.641^**^
Item 14	4.982 (1.333)	−1.512 (0.062)	2.200 (0.125)	0.352^**^	0.569^**^
Item 15	4.787 (1.475)	−1.524 (0.062)	1.861 (0.125)	0.406^**^	0.407^**^
PB ($\bar{\omega }$ = 88)	9.746 (6.148)	**2.344 (0.062)**	6.189 (0.125)	0.271^**^	
TB ($\bar{\omega }$ = 78)	24.33 (18.547)	1.120 (0.062)	1.417 (0.125)	0.132^**^	

*PB, Perceived Burdensomeness; TB, Thwarted Belongingness. Items 1–6 were correlated with the PB subscale; items 7–15 were correlated with the TB subscale. In bold, the skewness values higher than 2 and the kurtosis (proper) higher than 7 (51).*

a *Lilliefors significance correlation.*

** *p < 0.01*.

### Multi-Group Confirmatory Factor Analysis of the INQ-15

**Figure 1:**
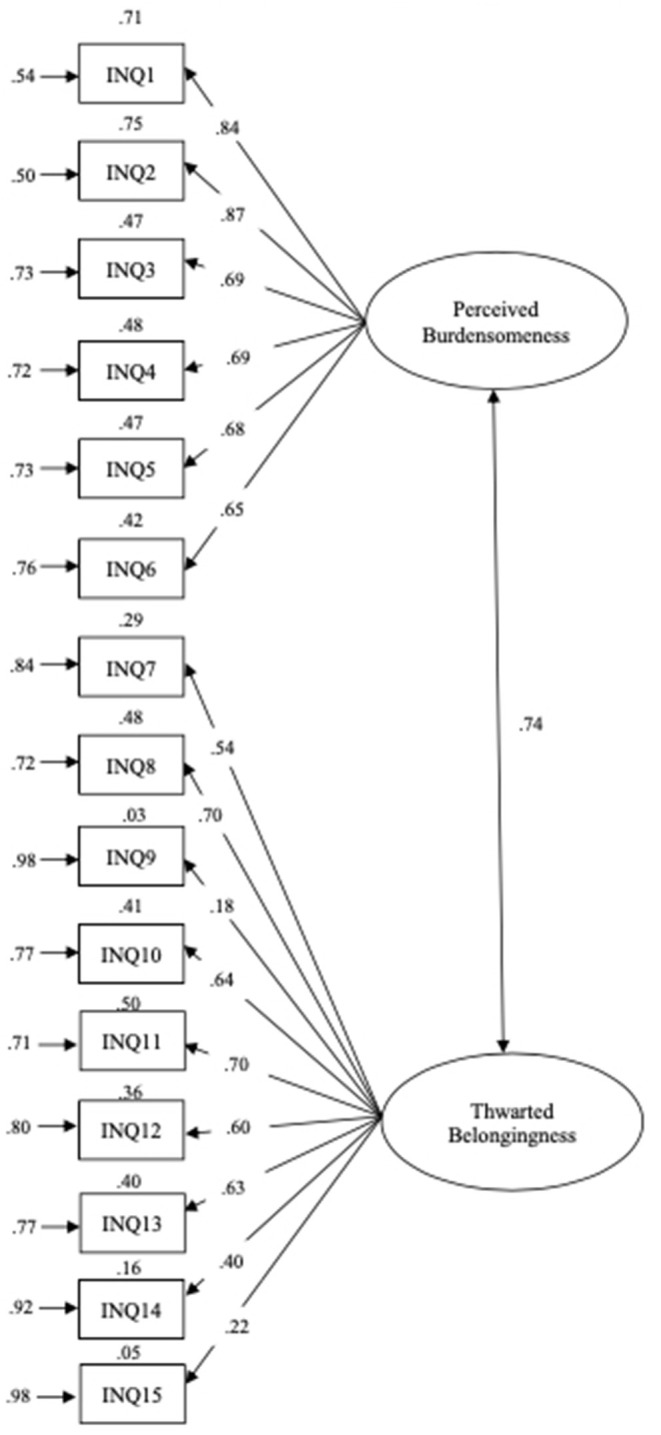
Model for the INQ-15 obtained in the present study. Values at the top of each rectangle are *R*^2^; values at the left of each rectangle are errors.

In the whole sample, the PB subscale showed a good fit, *SB*χ^2^_(9)_ = 6.448, *p* = 0.694, CFI = 1.000, RMSEA = 0.000 [90% CI (0.000, 0.022)], and the TB showed an acceptable fit, *SB*χ^2^_(27)_ = 248.973, *p* = 0.000, CFI = 0.922, RMSEA = 0.073 [90% CI (0.065, 0.082)]. All parameters were statistically significant at the 0.05 level (Figure 1).

Table 3 shows the baseline models for the boys' and girls' groups, as well as the analysis of invariance of the INQ-15 subscales across sex and age (if needed).

**Table 3:** Test of invariance across sex and age for the INQ-15 subscales.

	**Subscale**	**Invariance**	***SBχ*^2^ (*df*)**	***p***	**CFI**	**RMSEA (90% CI)**	***ΔSBχ*^2^ (Δ*df*)**	**ΔCFI**	**ΔRMSEA**
Sex	PB	Baseline boys	3.004 (9)	0.964	1.000	0.000 (0.000, 0.000)			
		Baseline girls	3.965 (9)	0.914	1.000	0.000 (0.000, 0.015)			
		Configural	6.969 (18)	0.990	1.000	0.000 (0.000, 0.000)			
		Metric	34.583 (24)	0.075	0.993	0.024 (0.000, 0.041)	27.614 (6)	0.007	**0.024**
	TB	Baseline boys	133.346 (27)	0.000	**0.890**	0.073 (0.061, 0.086)			
		Baseline girls	123.866 (27)	0.000	0.951	0.067 (0.055, 0.079)			
Age	PB	Baseline 12-15	2.614 (9)	0.978	1.000	0.000 (0.000, 0.000)			
		Baseline 16-19	4.647 (9)	0.864	1.000	0.000 (0.000, 0.025)			
		Configural	7.261 (18)	0.988	1.000	0.000 (0.000, 0.000)			
		Metric	14.204 (24)	0.942	1.000	0.000 (0.000, 0.006)	6.943 (6)	0.000	0.000
		Scalar	19.168 (29)	0.917	1.000	0.000 (0.000, 0.010)	4.964 (5)	0.000	0.000
		Strict	20.838 (35)	0.972	1.000	0.000 (0.000, 0.000)	1.670 (6)	0.000	0.000
	TB	Baseline 12-15	245.214 (27)	0.000	**0.881**	**0.091** (0.080, 0.101)			
		Baseline 16-19	42.493 (27)	0.029	0.985	0.032 (0.010, 0.050)			

*PB, Perceived Burdensomeness; TB, Thwarted Belongingness.*

*In bold, CFI < 0.90 and RMSEA > 0.080*.

#### PB Invariance

The PB subscale showed a good fit in the boys' group, CFI > 0.95, and an acceptable fit in the girls' group, CFI > 0.90. The RMSEA index was <0.050 for both sex groups. The configural model showed a good fit, CFI > 0.95 and RMSEA <0.050. For the metric invariance, although the ΔCFI was below 0.01, the ΔRMSEA above 0.015 suggested non-invariance. Regarding the age groups, the baseline and configural model was adequate for both the 12–15 and 16–19 year-old, and metric, scalar, and strict invariance were obtained, with ΔCFI and ΔRMSEA values below the recommended 0.01 and 0.015, respectively.

#### TB Invariance

In both boys and girls groups, the RMSEA was acceptable, with a value higher than 0.050, but lower than 0.080. The CFI > 0.95 was adequate for the girls' group, both the CFI <0.90 was inadequate for the boys' group (therefore, the invariance analysis was not performed). Regarding the age groups, the model was adequate for the 16–19 year-old group, CFI > 0.95 and RMSEA <0.050, but inadequate for the 12–15 year-old group, CFI <0.90 and RMSEA > 0.80 (therefore, the invariance analysis was not performed).

### Convergent and Discriminant Validity of the INQ-15

Table 4 shows the correlations between the INQ-15 subscales and the ISAS intrapersonal and interpersonal functions, NSSI frequency to determine convergent validity, and the PIL-A to determine discriminant validity. Regarding the convergent validity, PB and TB showed positive but low significant correlations with NSSI frequency and HS. In the case of discriminant validity, negative and significant moderate-high correlations were found between the PIL-A and both the PB and TB subscales from the INQ-15.

**Table 4:** Convergent and divergent validity of the INQ-15 subscales.

**INQ-15** **subscales**	**Whole sample (*n* = 1,536)**			**Boys (*n* = 739)**			**Girls (*n* = 797)**			**Comparison of boys' and girls'** **correlations (*Z*)**			**Effect size for the difference** **between the boys' and** **girls' correlations^a^**		
	**HS**	**NSSI**	**PIL-A**	**HS**	**NSSI**	**PIL-A**	**HS**	**NSSI**	**PIL-A**	**HS**	**NSSI**	**PIL-A**	**HS**	**NSSI**	**PIL-A**
PB	0.26^***^	0.26^***^	−0.49^***^	0.43^***^	0.22^***^	−0.43^***^	0.51^***^	0.30^***^	−0.55^***^	−2.01^*^	−1.68^*^	3.10^***^	0.103 (S)	0.086 (N)	0.158 (S)
TB	0.24^***^	0.24^***^	−0.51^***^	0.42^***^	0.21^***^	−0.45^***^	0.47^***^	0.27^***^	−0.56^***^	−1.22	−1.25	2.90^**^			0.148 (S)

*PB, Perceived Burdensomeness; TB, Thwarted Belongingness; HS, Hopelessness; NSSI, Non-Suicidal Self-Injuries; PIL-A, Purpose in Life-Adolescents; (S), Small effect; (N), Non effect.*

a *Cohen (49).*

* *p < 0.05.*

** *p < 0.01.*

*** *p < 0.001*.

The differences between boys' and girls' correlations and the effect-sizes of these differences were calculated. The correlations between both PB and TB and HS, NSSI frequency, and the PIL-A were higher for girls, and the differences between girls and boys were significant for the correlations between the PB subscale and HS, NSSI frequency, and the PIL-A, as well as for the correlation between TB and the PIL-A. Of these differences, the PB-HS, PB-PIL-A, and TB-PIL-A showed a small effect-size, and the PB-NSSI showed no effect, according to Cohen (49).

## Discussion

The main aims of the present study were: (1) to translate and confirm the internal consistency and two-factor structure of the INQ-15 in a Spanish sample of community adolescents between 12 and 19 years old; (2) to analyze the invariance of the INQ-15 across sex and age (12–15 and 16–19 year-olds); and (3) to analyze the validity of the scale.

We obtained good internal consistency for both the PB and TB subscales, $\bar{\omega }$ = 0.88 and $\bar{\omega }$ = 0.78, respectively, which are similar to those obtained in previous studies with college students [e.g., (30)] and lower than those obtained in other studies with adult psychiatric outpatients [e.g., (52)] and adolescents between 12 and 17 years old (32). Moreover, the results obtained in the present study support the factorial validity of the two-factor INQ-15, as proposed and confirmed by Van Orden et al. (19) and Nademin et al. (20): six items make up PB (1–6), and nine items form TB (7–15). Van Orden et al. (19) confirmed the factorial structure of the INQ-15 in undergraduate students, adult outpatients, and older adults. Nademin et al. (20) confirmed it in military personnel, and Hill et al. (30) in college students. However, the two studies that analyzed the factorial structure of the INQ-15 in adolescents did not confirm this 15-item structure (30, 32). Podlogar et al. (32) referred to the wording valence rather than construct overlap to explain their results. In our study, we did not find wording problems, and all the items properly represented the two constructs. Thus, this study supports the 15-item version in Spanish adolescents, thus adding to the instruments available to screen risk factors for suicide in Spain.

As for the structure validity of the INQ-15 subscales according to sex and age, the model for the PB subscale was adequate for boys and girls, as well as for both age groups. The model for the TB subscale was adequate only for the girls and the 16–19 year-old group. Because the model for the TB subscale was inadequate in the boys' group, the invariance analysis was carried out only for the PB subscale. The configural model showed good values. Regarding the metric invariance, although the ΔCFI was notably lower than the recommended 0.01, the ΔRMSEA was higher than 0.015 (47). Together [Cfr. (53)], these results indicate that the INQ-15 subscales did not show invariance across sex. Therefore, we could not analyze the differences in the PB and TB scales according to sex [e.g., (54, 55)]. Future studies should analyze the invariance in the INQ-15 across sex, to carry out further analyses of sex-related differences in the PB and TB subscales.

Because the model for the TB subscale was inadequate in the group of 12–15-year-old, the invariance analysis was carried out only for the PB subscale and all the estimated standardized factor loadings were significant.

Regarding convergent validity, PB and TB showed positive, significant correlations with frequency of NSSI and hopelessness. These associations show that the two factors of the IPTS, the perception of being a burden and loneliness and the perception of not receiving any kind of reciprocal care, are related to other factors included in the theory (hopelessness), but also to other variables classically linked to suicide, such as NSSI [i.e., (56, 57)] supporting the validity of the INQ-15 for assessing suicide-like constructs. Likewise, PB and TB showed negative associations with the protective factor meaning in life. That is, the more the adolescent perceives that her/his life makes sense, is meaningful and trustful, and is worth living, the less he/she feels like a burden to others and thinks his/her belongingness is altered or lacking.

Finally, we found positive associations between PB, hopelessness, and the frequency of NSSI, as well as negative associations between PB and TB and between TB and meaning in life, but they were stronger in girls than in boys. Although the simple correlations do not allow us to draw conclusions about the nature of these differences, these results could point to sex-related differences in the PB and TB subscales (54, 55) and in the relationships between these subscales and other variables. Future studies on this issue are needed.

### Strengths and Clinical Implications

Although several studies have explored the factorial structure and psychometric properties of the INQ-15 [i.e., (19, 21)], this is the first study to confirm the factorial structure of the INQ-15 in a Spanish sample of community adolescents. The results support the validity of the instrument for assessing suicide-like constructs in Spain, where this is an important problem in young populations that requires valid and reliable measures. In addition, we used a broad sample that allowed us to test the factorial structure of the instrument.

### Limitations and Future Research

The current study has some limitations. First, data distribution was not normal for several items and the PB subscale of the INQ-15. Second, due to the lack of a re-test measurement, we cannot corroborate the temporal stability of the Spanish version of the INQ-15. In addition, because this study is part of a broader study of NSSI in Spanish adolescents, we did not examine the convergent validity of the INQ-15 with other validated measures of suicide-like behaviors, such as suicide ideation or attempts, which is an important flaw in this study. Thus, future longitudinal studies are needed to examine the predictive power of PB and TB in the occurrence of suicide plans and attempts in Spanish adolescents.

## Conclusion

Our results suggest that it is appropriate to use the INQ-15 to assess PB and TB in Spanish adolescents from 12 to 19 years old. However, future research should specifically revise the model fit of the INQ-15 in boys and adolescents between 12 and 15 years old, as well as its invariance across sex and age.

## Data Availability Statement

The raw data supporting the conclusions of this article will be made available by the authors, without undue reservation.

## Ethics Statement

The studies involving human participants were reviewed and approved by Ethics Committee of the Catholic University of Valencia, Research Code UCV2015-2016/0025-V2. Written informed consent to participate in this study was provided by the participants' legal guardian/next of kin.

## Author Contributions

SP: design, verify evolution of research, data curation, data analyses, writing original draft, writing review and editing the manuscript, and project administration. JG-A: data curation, data analyses, writing original draft, and writing review and editing the manuscript. BG: sample assessment, data analyses, and writing review and editing the manuscript. VG: data analyses and review and editing the manuscript. JM: design, verify evolution of research, data curation, data analyses, writing review and editing the manuscript, and project administration. All authors contributed to the article and approved the submitted version.

## Funding

Funding for the study was provided by R + D + I Projects of the State Programs Oriented to the Challenges of Society, within the framework of the State Research Plan Scientific and Technical and Innovation, with Code: PID2019-111036RB-I00, from Ministry of Science and Innovation of Spain.

## Conflict of Interest

The authors declare that the research was conducted in the absence of any commercial or financial relationships that could be construed as a potential conflict of interest.

## Publisher's Note

All claims expressed in this article are solely those of the authors and do not necessarily represent those of their affiliated organizations, or those of the publisher, the editors and the reviewers. Any product that may be evaluated in this article, or claim that may be made by its manufacturer, is not guaranteed or endorsed by the publisher.
